# Impact of PCB and *p*,*p*′-DDE Contaminants on Human Sperm Y:X Chromosome Ratio: Studies in Three European Populations and the Inuit Population in Greenland

**DOI:** 10.1289/ehp.8668

**Published:** 2005-12-29

**Authors:** Tarmo Tiido, Anna Rignell-Hydbom, Bo A.G. Jönsson, Yvonne Lundberg Giwercman, Henning S. Pedersen, Bogdan Wojtyniak, Jan K. Ludwicki, Vladimir Lesovoy, Valentyna Zvyezday, Marcello Spano, Gian-Carlo Manicardi, Davide Bizzaro, Eva C. Bonefeld-Jørgensen, Gunnar Toft, Jens Peter Bonde, Lars Rylander, Lars Hagmar, Aleksander Giwercman, INUENDO

**Affiliations:** 1 Molecular Reproductive Medicine Research Unit, Department of Clinical Sciences, Fertility Centre, Malmö University Hospital, Lund University, Malmö, Sweden; 2 Department of Occupational and Environmental Medicine, Lund University Hospital, Lund, Sweden; 3 Department of Urology, Malmö University Hospital, Lund University, Malmö, Sweden; 4 Centre for Arctic Environmental Medicine, Nuuk, Greenland; 5 Department of Medical Statistics and; 6 Department of Environmental Toxicology, National Institute of Hygiene, Warsaw, Poland; 7 Regional Clinical Center of Urology and Nephrology, Kharkiv, Ukraine; 8 Laboratory of Human Reproduction, Kharkiv State Medical University, Kharkiv, Ukraine; 9 Section of Toxicology and Biomedical Sciences, BIOTEC-MED, ENEA Casaccia Research Centre, Rome, Italy; 10 University of Modena and Reggio Emilia, Modena, Italy; 11 Istituto di Biologia e Genetica Università Politecnica delle Marche, Ancona, Italy; 12 Institute of Public Health, Unit of Cellular and Molecular Toxicology, Department of Occupational and Environmental Medicine, University of Aarhus, Aarhus, Denmark; 13 Department of Occupational Medicine, Aarhus University Hospital, Aarhus, Denmark

**Keywords:** polychlorinated biphenyls, POP, *p, p′*-DDE, sex chromosomes, sex ratio, sperm

## Abstract

**Objective:**

Recent studies indicate that persistent organohalogen pollutants (POPs) may contribute to sex ratio changes in offspring of exposed populations. Our aim in the present study was to investigate whether exposure to 2,2′,4,4′,5,5′-hexachlorobiphenyl (PCB-153) and dichlorodiphenyldichloroethene (*p*,*p*′-DDE) affects sperm Y:X chromosome distribution.

**Subjects and methods:**

We obtained semen and blood for analysis of PCB-153 and *p*,*p*′-DDE levels from 547 men from Sweden, Greenland, Poland (Warsaw), and Ukraine (Kharkiv), with regionally different levels of POP exposure. The proportion of Y- and X-chromosome–bearing sperm in the semen samples was determined by two-color fluorescence *in situ* hybridization analysis.

**Results:**

Swedish and Greenlandic men had on average significantly higher proportions of Y sperm (in both cohorts, 51.2%) and correspondingly higher lipid-adjusted concentrations of PCB-153 (260 ng/g and 350 ng/g, respectively) compared with men from Warsaw (50.3% and 22 ng/g) and Kharkiv (50.7% and 54 ng/g). In the Swedish cohort, log-transformed PCB-153 and log-transformed *p*,*p*′-DDE variables were significantly positively associated with Y-chromosome fractions (*p*-values 0.04 and < 0.001, respectively). On the contrary, in the Polish cohort PCB-153 correlated negatively with the proportion of Y-bearing fraction of spermatozoa (*p* = 0.008).

**Conclusions:**

The present study indicates that POP exposure might be involved in changing the proportion of ejaculated Y-bearing spermatozoa in human populations. Intercountry differences, with different exposure situations and doses, may contribute to varying Y:X chromosome ratios.

Recent studies have indicated that the proportion of male births has been declining in many countries during the past five decades (Allan et al. 1997; Marcus et al. 1998; Møller 1998; Parazzini et al. 1998; van der Pal-de Bruin et al. 1997). The cause of such a time-related trend is not known but has been suggested to result from an increasing exposure to endocrine disruptors such as persistent organohalogen pollutants (POPs) (Toppari et al. 1996).

POPs—for example, polychlorinated dibenzofurans (PCDFs), polychlorinated dibenzo-*p*-dioxins (PCDDs), polychlorinated biphenyls (PCBs), dichlorodiphenyl-trichloroethane (DDT), and dichloro-diphenyldichloroethene (*p*,*p*′-DDE), the most stable metabolite of DDT—are ubiquitous environmental contaminants. Because of the hydrophobic and lipophilic nature and their long half-lives, these compounds are highly persistent and have a tendency to bio-accumulate and biomagnify in the food chain. Studies have shown that measurable levels of PCBs and *p*,*p*′-DDE are found in a large proportion of the general population [Arctic Monitoring and Assessment Programme (AMAP) 2003; Longnecker et al. 1997].

Some of these POPs can disrupt multiple endocrine pathways and induce a wide range of toxic responses (Toppari et al. 1996). A number of studies have demonstrated their estrogenic, antiestrogenic, dioxin-like, and androgen-competing properties (Andersen et al. 2002; Bonefeld-Jørgensen et al. 2001; Danzo 1997). Dioxin toxicity is most potent when animals are exposed *in utero* and lactationally. Although single exposure to dioxin has been investigated in a number of studies, there are few reports on repeated exposure of low dioxin doses, which more resembles the human situation. Recently, Ikeda et al. (2005) showed that *in utero* and lactational exposure of male rats to dioxin decreased the sex ratio of the subsequent generation. With respect to human exposure, two accidents that have attracted scientific and public attention are the Yucheng poisoning (Chen et al. 1985; Masuda et al. 1985) and the Seveso disaster (Mocarelli et al. 1996), both of which were associated with an increased proportion of girls born subsequent to paternal exposure to POPs (del Rio Gomez et al. 2002; Mocarelli et al. 1996, 2000). In human populations exposed to more moderate levels of POPs, both increased (Karmaus et al. 2002) and decreased (Rylander et al. 1995) male:female sex ratios have been reported. Therefore, the explanation of the secular trend in sex ratio is still lacking, and the mechanisms that can affect the proportion of males to females are not yet understood.

Theoretically, offspring sex ratio may be related to events that occur before fertilization that favor selection of Y- or X-chromosome–bearing spermatozoa, events that occur after fertilization such as preferential development or survival of embryos of one sex, or a combination of both. Although recent human studies have indicated that paternal exposure to POPs has a deleterious effect on some semen characteristics (Guo et al. 2000; Hauser et al. 2003; Richthoff et al. 2003; Rignell-Hydbom et al. 2005), it is not yet known whether these compounds could change the proportion of X- and Y-bearing sperm.

Recently, in a population composed of Swedish fishermen, we found a moderate positive association between serum levels of PCB-153 and of *p*,*p*′-DDE and the proportion of Y-bearing spermatozoa (Tiido et al. 2005). The study was part of a European Union–supported collaboration (INUENDO 2006) aiming to enlighten the impact of POP exposure on human reproductive function. Other populations included in this collaboration were recruited from Greenland, Poland (Warsaw), and Ukraine (Kharkiv). We have chosen to use the PCB congener, 2,2′,4,4′,5,5′-hexachloro-biphenyl (PCB-153) as a biomarker for POP exposure because of its very high correlations with the total PCB concentration (Glynn et al. 2000; Grimvall et al. 1997), the estimated 2,3,7,8-tetrachlorodibenzo-*p*-dioxin (TCDD) equivalent (TEQ) from PCB, and the total POP-derived TEQ (Gladen et al. 1999), respectively. Likewise, the major DDT metabolite *p*,*p*′-DDE, an antiandrogenic compound, is another good indicator of the exposure. Previous studies from Greenland, Ukraine, and Sweden indicate that the exposure levels for *p*,*p*′-DDE are still considerable (Deutch et al. 2004; Gladen et al. 1999; Sjödin et al. 2000).

The aim of the present study was to investigate whether the previously reported positive association between POP exposure markers and the proportion of Y-bearing sperm also occurs in three other populations characterized by different POP exposure profiles than the one found among Swedish fishermen (Jönsson et al. 2005). This information might indirectly add to our understanding of the biologic link between POP exposure and offspring sex ratio.

## Materials and Methods

### Study population.

We recruited subjects in the four participating countries: Greenland, Poland (Warsaw), Ukraine (Kharkiv), and Sweden.

In Sweden, we obtained semen and blood samples from 191 professional fishermen from the Swedish east and west coasts cohorts that participated in semen study between March 2001 and November 2001, and March 2002 and September 2002. Initially, 2,783 Swedish fishermen had been informed about the semen study, and 266 (10% participation rate) gave their written informed consent to participate. However, 75 subjects had to be excluded during the sampling period because of logistical reasons, changes of mind, sickness, or recent vasectomy during the field study period. Details regarding the study population were reported previously (Rignell-Hydbom et al. 2004). Of the participants, 79.5% had sired a child (or children). Circumstantial evidence based on data from the Swedish Medical Birth Register provided support that there was no difference in number of fathered children between participants and nonparticipants. The data on the Swedish fishermen are identical to those previously reported (Tiido et al. 2005).

In the other three countries, consecutive pregnant women were approached as the entry point for the study, and their male spouses were enrolled in the study from May 2002 throughout February 2004. For inclusion, both the man and his spouse had to be at least 18 years of age.

In Greenland, we asked 256 male partners of pregnant women to participate in the study. All were of Inuit origin. In Warsaw, we enrolled altogether 690 spouses of pregnant women, who visited either the obstetric out-patient clinic of the Gynaecological and Obstetric Hospital of the Warsaw School of Medicine or physicians at a collaborating hospital in the same city. In Kharkiv, 640 male spouses of pregnant women who visited one of eight antenatal clinics or three maternity hospitals were informed about the project and asked to participate. Blood and semen samples were collected, and 201 men from Greenland (79% participation rate), 198 from Warsaw (29% participation rate), and 208 from Kharkiv (33% participation rate) were interviewed.

**Figure 1:**
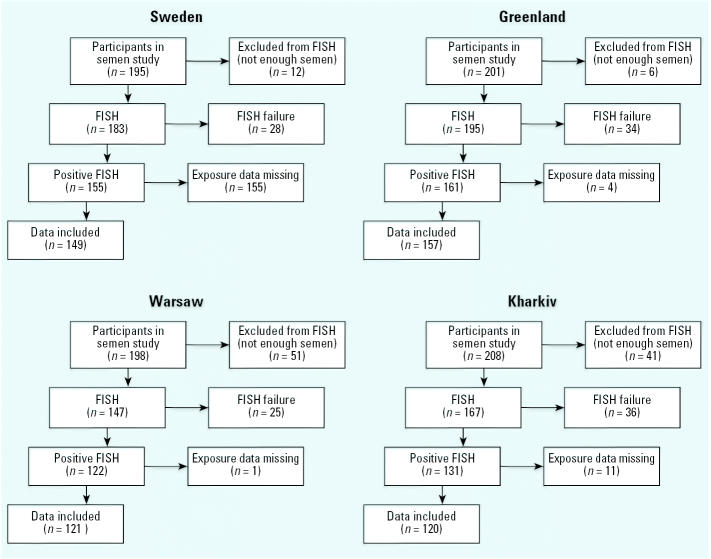
Flow chart for recruitment of participants in semen and FISH study.

For 110 men, not enough semen was available for fluorescence *in situ* hybridization (FISH) analysis, but we made attempts to determine the Y- and X-chromosome fractions in spermatozoa by FISH analysis for the remaining 692 subjects (Figure 1). However, FISH analyses were successfully performed for 569 men. We excluded the remaining 123 samples because of low number of cells available or failure during analysis. We found no statistically significant differences regarding age, lipid-adjusted levels of PCB-153 and *p*,*p*′-DDE, percentage A+B motile sperm (except Greenland, where mean value was higher in participants than in nonparticipants, *p* = 0.03), or sperm concentration (except Kharkiv, where concentrations were higher in participants, *p* < 0.001) between the participating men and the subjects who were excluded because of low number of cells available or hybridization failure. Exposure data were lacking for 22 men; the final results are thus based on 547 subjects.

The study was approved by local ethical committees representing all participating populations, and all subjects signed an informed consent.

### Semen and blood sampling, and questionnaire.

Semen samples were collected by masturbation at the participant residence (Sweden and Greenland) or in privacy in a room at the hospital (Warsaw and Kharkiv). We asked the subjects to abstain from sexual activities for at least 48 hr before collecting the sample and to note the actual abstinence time.

The sample was kept close to the body to maintain a temperature close to 37°C when transported to the laboratory immediately after collection. Two Nunc cryotubes (VWR International, Roskilde, Denmark) with 0.2-mL aliquots of undiluted raw semen, collected 30 min after liquefaction, were prepared from each semen sample, coded, and directly put on dry ice or frozen in −20°C and transferred to −80°C within 2 weeks. In addition, we analyzed the samples for concentration, motility, and morphology (Toft et al. 2005) using methods described by the World Health Organization (1999). Venous blood samples were collected within 1 week of the semen collection, except for a subgroup of 116 Greenlandic samples, which were collected up to 1 year in advance. The blood samples were centrifuged immediately after collection, and sera were stored at −80°C for subsequent analysis.

We collected information on lifestyle (alcohol consumption and smoking habits) through interviews. Participants from the three cohorts of partners of pregnant women (Greenland, Warsaw, Kharkiv) were contacted after the expected time of delivery in order to get information about the pregnancy outcome and the child’s sex.

The background characteristics of the study populations are presented in Table 1. Sperm characteristics of the Swedish population have previously been reported (Rignell-Hydbom et al. 2004). There were no cases with azoospermia. Sperm concentration ranged between 3.3 and 419 × 10^6^/mL, and the median was 59 × 10^6^/mL (Sweden: 5.7–207 × 10^6^/mL, median 50.1 × 10^6^/mL; Greenland: 10–374 × 10^6^/mL, median 57 × 10^6^/mL; Kharkiv: 6.6–320 × 10^6^/mL, median 65 × 10 ^6^/mL; Warsaw: 3.3–419 × 10^6^/mL, median 73 × 10^6^/mL).

### Two-color FISH and scoring criteria.

Preparation of sperm, *in situ* hybridization, and determination of scoring criteria were essentially as described in Tiido et al. (2005). Briefly, we decondensed sperm heads on slides by incubation in 10 mM dithiothreitol (Saveen Werner AB, Malmö, Sweden)/0.1 M Tris for 7 min, followed by incubation in 1 mM DDT/4 mM lithium diiodosalicylate (Sigma-Aldrich Chemie GmbH, Steinheim, Germany)/0.1 M Tris for 20 min. Slides were then washed in 2× saline-sodium citrate (SSC) and air dried. Thereafter, we accomplished the hybridization using protein-nucleic acid probes (provided by DakoCytomation, Glostrup, Denmark) targeted against the centromeric region of the X chromosome (rhodamine-labeled) and the q-arm of the Y chromosome (labeled with fluorescein isothiocyanate). The probe mixture was placed on the semen smears, mounted with a cover slip, and sealed with rubber cement. Subsequently, probe and target DNA were denatured; after overnight hybridization at 37°C, slides were washed two times, 5 min each, at 36°C in 60% formamide/2× SSC, in 0.2× SSC for 5 min at room temperature, and 5 min in Tris-HCl/ NaCl-buffer/0.05% Tween 20. Slides were rinsed in 2× SSC and dehydrated in an ethanol series (70, 90, 100%). Thereafter, the slides were counterstained with 0.1 μg/mL of 4′,6-diamidino-2-phenylindole for 30 sec and dehydrated. Finally, we mounted the slides in Vectashield antifade medium (Vector Laboratories Inc., Burlingame, CA, USA). Thereafter, the microscopic examination was performed blindly, that is, without knowledge of the exposure levels or other subject characteristics. An X or Y chromosome in a sperm nucleus was recognized by a red or a green fluorescent spot, respectively. In every sample, the proportion of sperm presenting with a clear red or green signal was ≥95%.

The Y/X-chromosome status of the spermatozoa was evaluated by assessing randomly selected visual fields. In the Swedish cohort, we evaluated between 276 and 1,301 cells. This variation was due to a quality control program that was included in this part of the study in order to assess the interobserver and intraobserver coefficient of variation (CV). We estimated that interobserver and intraobserver CVs with respect to the proportion of Y-bearing sperm were 2.3% and 3.3%, respectively, by scoring 500 cells only; this procedure was subsequently to be applied to other samples. However, because of the quality of the slides for six Swedish samples, < 500 nuclei were scored (4% of samples with < 500 nuclei). The median number of cells counted in remaining subjects was 524 (range, 268–593; 35% of samples with < 500 nuclei) in Greenland, 537 (range, 304–743; 17% of samples with < 500 nuclei) in Warsaw, and 488 (range, 253–594; 54% of samples with < 500 nuclei) in Kharkiv.

### Determination of PCB-153 and p,p′-DDE in serum.

We performed all analyses of PCB-153 and *p*,*p*′-DDE in serum at the Department of Occupational and Environmental Medicine in Lund, Sweden, applying solid-phase extraction using on-column degradation of the lipids and analysis by gas chromatography–mass spectrometry as previously described (Richthoff et al. 2003; Rignell-Hydbom et al. 2004). Levels of detection, CVs, and participation in quality control programs have been described in detail elsewhere (Jönsson et al. 2005).

### Determination of lipids by enzymatic methods.

Serum concentrations of triglycerides and cholesterol were determined by enzymatic methods as described elsewhere (Jönsson et al. 2005). The total lipid concentration in serum (grams per liter) was calculated by the following equation (Rylander et al. 2006):


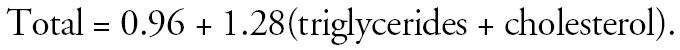


### Statistical analysis.

We used SPSS software (SPSS for Windows 12.0; SPSS Inc., Chicago, IL, USA) for statistical analyses; the level of significance was set at *p* ≤0.05.

To evaluate the effect of the exposure variables PCB-153 and *p*,*p*′-DDE on the fraction of Y chromosomes, we performed linear regression model analysis. PCB-153 and *p*,*p*′-DDE data were analyzed as continuous variables (untransformed and log transformed) as well as five arbitrarily categorized groups (0–50, > 50–100, > 100–200, > 200–400, and > 400 ng/g lipid for PCB-153; 0–250, > 250–500, > 500–1,000, > 1,000–1,500, and > 1,500 ng/g lipid for *p*,*p*′-DDE). We checked model assumptions by means of residual analyses. Because PCB-153 and *p*,*p*′-DDE serum levels were highly correlated in Inuit and Swedish fishermen (*r* = 0.93 and 0.79, respectively) (Jönsson et al. 2005), both variables were not taken into the models simultaneously. For evaluation of heterogeneity, we included an interaction term (exposure × study population) in the linear regression test. If this term was significant, we performed separate analyses for each population.

Furthermore, the whole study population was divided into groups with four different exposure profiles, dependent on whether the PCB-153 and *p*,*p*′-DDE levels were above (high) or below (low) the median for all men included in the study. The proportion of the Y-bearing sperm was compared in the four exposure profile groups (high PCB-153/low *p*,*p*′-DDE, high PCB-153/high *p*,*p*′-DDE, low PCB-153/low *p*,*p*′-DDE, low PCB-153/ high *p*,*p*′-DDE) by use of statistical models as described above. This analysis was also preceded by evaluation of heterogeneity between the study population and the exposure profile group (interaction term: exposure profile group × study population).

Based on information from the literature (Fukuda et al. 2002; Hilsenrath et al. 1997), we considered the following covariates as potential confounders: age (as a continuous variable or categorized as < 30, 30–45, and > 45 years), period of sexual abstinence before delivery of the semen sample (as log-transformed continuous variable or categorized into 0–2, > 2–4, > 4–6, > 6 days), and smoking (current smoking status; yes/no). Moreover, alcoholic beverage consumption (> 21 drinks/week; yes/no) was used. These potential confounders were listed *a priori* and were evaluated according to the change-inestimate method (Greenland 1989), which includes confounders in the final model only if they change the effect estimate by > 10% and excludes them again if exclusion change the effect estimate by < 5%.

For 457 of the men recruited from Greenland, Warsaw, and Kharkiv, the outcome of the pregnancy associated with the current sample collection was known. We compared the proportion of boys for the quartile with the highest percentage of Y-sperm to the one with the lowest percentage of Y-bearing spermatozoa.

## Results

### Intercohort differences in proportions of Y-bearing sperm.

**Figure 2:**
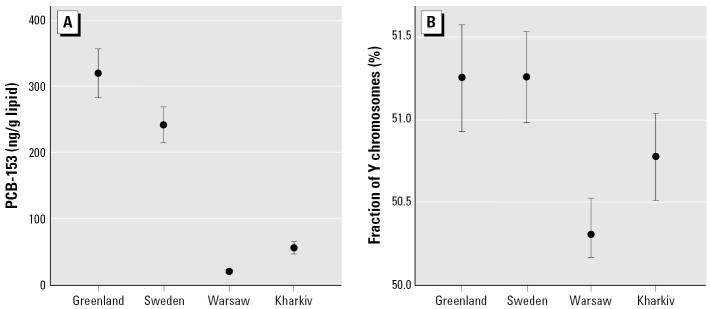
Distribution of PCB-153 (ng/g lipid) serum levels (*A*) and fraction of Y-bearing sperm in ejaculates (*B*) among men from different countries studied. Values shown are means and 95% CIs.

The highest proportions of Y-chromosome fractions were found among Inuit and Swedish fishermen, whereas the lowest were noted in men from Kharkiv and Warsaw (Table 1, Figure 2). There was no difference between the Swedish and the Greenlandic cohorts [crude mean difference, 0.006%; 95% confidence interval (CI), −0.38 to 0.37; *p* = 0.97]. This was also evident after adjustment for age, period of sexual abstinence, and serum PCB-153. The fishermen from Sweden and the Greenland Inuit had higher fractions of Y-bearing sperm compared with the men from Kharkiv [adjusted mean difference, 0.67% (95% CI, 0.04–1.31; *p* = 0.03) and 0.47% (95% CI, 0.02–0.92; *p* = 0.04), respectively] and Warsaw [adjusted mean difference, 1.1% (95% CI, 0.44–1.65; *p* = 0.001) and 0.84% (95% CI, 0.35–1.33; *p* = 0.001), respectively]. Men from Kharkiv in turn had a similar proportion of Y-sperm as men from Warsaw (adjusted mean difference, 0.37%; 95% CI, −0.12 to 0.87; *p* = 0.14). The relative magnitude of the proportion of Y-sperm corresponded to the pattern of the average lipid-adjusted concentrations of PCB-153, with higher mean values in Inuit men (320 ng/g lipid) and Swedish fishermen (240 ng/g lipid) compared with men both from Warsaw (19 ng/g lipid) and Kharkiv (55 ng/g lipid) (Figure 2). In contrast, the pattern of mean Y-chromosome fractions with respect to country did not correspond to the mean levels of *p*,*p*′-DDE (data not shown).

### PCB-153 and p,p′-DDE levels versus proportion of Y-bearing sperm.

Crude proportions of Y-bearing spermatozoa according to serum PCB-153 and *p*,*p*′-DDE concentration in the study groups are shown in Tables 2 and 3, respectively.

For PCB-153, and especially for *p*,*p*′-DDE, the log-transformed variables better fulfilled model assumptions compared with the untransformed ones. Although confounder-adjusted sperm Y-chromosome fractions were unrelated to log-transformed lipid-adjusted PCB-153 concentration among the Inuit men (β= 0.03; 95% CI, −0.33 to 0.41; *p* = 0.83) and men from Kharkiv (β= 0.23; 95% CI, −0.18 to 0.64; *p* = 0.27), increasing PCB-153 serum levels were associated with increasing proportions of Y-bearing sperm in the Swedish cohort (β= 0.53; 95% CI, 0.001–1.05; *p* = 0.04). Among men from Warsaw, however, the association was negative (β= −0.54; 95% CI, −0.92 to −0.14; *p* = 0.008) (Table 4). According to the regression model in the Swedish cohort, this means that the PCB-153 concentration of 200 ng/g lipid (the median level) corresponded to a Y-chromosome fraction of 51.7% and that the concentration of 401 ng/g lipid (lower limit value for highest exposure category) corresponded to 52.1%.

The log-transformed lipid-adjusted *p*,*p*′-DDE concentration was significantly (*p* < 0.001) associated with the Y-chromosome fraction (β= 0.66; 95% CI, 0.30–1.02) in Swedish fishermen but not in any other group (Table 4).

When we divided PCB-153 and *p*,*p*′-DDE levels into five categories (Table 5), there were no statistically significant differences in proportion of Y-bearing sperm between the group with the highest levels of PCB-153 and *p*,*p*′-DDE and the one with the lowest levels. However, because of cohort-to-cohort differences in exposure levels, such comparisons were not possible for both exposures in all cohorts (Tables 2, 3, and 5).

The interaction between the exposure profile group (PCB-153/*p*,*p*′-DDE: high/low, high/high, low/low, low/high) and study population was not statistically significant (*p* = 0.17). We found statistically significant differences in the proportion of Y-bearing sperm between the group with low *p*,*p*′-DDE/ high PCB-153 and the group with high *p*,*p*′-DDE/low PCB-153 (adjusted mean difference, 0.75%; 95% CI, 0.23–1.27; *p* = 0.004). No significant differences were seen when low *p*,*p*′-DDE/low PCB-153 was compared with high *p*,*p*′-DDE/high PCB-153 (adjusted mean difference, 0.006%; 95% CI, −1.03, 1.02; *p* = 0.99).

### Sperm sex chromosome ratio versus offspring sex ratio.

In the joint material from Greenland, Warsaw, and Kharkiv, a comparison of the quartiles with the highest (53%) and the lowest (49%) proportion of Y-bearing sperm did not disclose any difference in the proportion of boys fathered by these men (53.4 vs. 52.6%; *p* = 0.91).

## Discussion

The main result of the present study was a positive association between PCB-153 and *p*,*p*′-DDE levels and the proportion of Y-bearing sperm in the Swedish cohort and the opposite trend with respect to PCB-153 among the men from Warsaw. With respect to *p*,*p*′-DDE, we found a statistically significant association among the Swedish fishermen but not in any of the other groups. Additionally, we found the highest mean percentages of Y sperm in the two cohorts (Greenlandic Inuit and Swedish fishermen) characterized by the highest levels of PCB-153 exposure. Thus, extension of the analysis to the additional three cohorts did not confirm the findings in our recent publication (Tiido et al. 2005) based on the Swedish fishermen.

Although the findings of this study might appear somewhat conflicting, such a pattern corresponds to the wide range of findings in epidemiologic studies evaluating the effect of different POPs on the offspring sex ratio outcome. Accidental high paternal TCDD exposure in the Seveso disaster significantly lowered the sex ratio among the offspring several years later (Mocarelli et al. 1996, 2000), and a similar trend was found after the Yucheng PCB and PCDF poisonings (del Rio Gomez et al. 2002). However, the more long-term but lower level of dioxin exposure among U.S. Vietnam veterans resulted in a tendency toward an increased proportion of boys fathered (Michalek et al. 1998). Similarly, among Michigan fish eaters, Karmaus et al. (2002) observed a positive association between paternal serum PCB concentrations and increased odds ratio of fathering a boy. On a cohort basis, families of Baltic Sea fishermen from the Swedish east coast had higher POP levels in blood than did families of fishermen from the west coast (Rylander and Hagmar 1999; Svensson et al. 1995). The proportion of boys fathered was significantly lower among men from the east coast cohort compared with the west coast cohort. None of these figures differed significantly, however, from the sex ratio of the general Swedish population (Rylander et al. 1995).

On the assumption that our associations observed in the present study are not chance findings, the differences between the four cohorts might be due to any of the following factors or a combination of them. First of all, there are differences in the POP exposure profiles among the four regions (Jönsson et al. 2005), the Inuit exhibiting high concentrations of both PCB-153 and *p*,*p*′-DDE, but *p*,*p*′-DDE levels are not as high as those in Kharkiv. In Kharkiv, however, the PCB-153 levels were low. The Swedish fishermen were somewhat less exposed to PCB-153 and *p*,*p*′-DDE compared with the Inuit, and the lowest exposure to PCB-153 and intermediate levels of *p*,*p*′-DDE were found in the Warsaw population (Jönsson et al. 2005). Apart from the differences regarding the PCB-153:*p*,*p*′-DDE ratio and absolute serum levels of these two chemicals in the four populations studied, we cannot exclude that the pattern of exposure to other POPs might also be subject to significant intercountry variation.

Second, although it is unknown whether the changes of sperm Y:X ratios are due to the hormone-like action of POPs, it is plausible that differing sex-hormone–mimicking actions of the various POPs contribute to the diverging effects. PCBs and pesticide residues may possess different types of sex-hormone agonistic and antagonistic activity (Bonefeld-Jørgensen et al. 2001). Furthermore, a number of POPs (e.g., coplanar PCBs and dioxins) are known to activate the aryl hydrocarbon receptor (AHR) (Pocar et al. 2005). We hypothesize that selective elimination of X-bearing germ cells for unknown reasons in the course of spermatogenesis may lead to Y:X changes in the pool of ejaculated spermatozoa. For example, abnormal meiosis at the spermatocyte stage and after elimination may be involved. Changes in offspring sex ratio after 1,2-dibromo-3-chloropropane (DBCP) exposure have been reported (Potashnik et al. 1984). A possible explanation was suggested to be Y-chromosome nondisjunction, being more frequent in sperm of DBCP workers (Goldsmith et al. 1984). It remains to be elucidated whether there might be a link between the sex-steroid or AHR pathways and meiotic disturbances. Because both the sex-hormone–dependent and the AHR-regulated pathways may be involved in the regulation of sex chromosome status of spermatozoa, the differences in the association between PCB-153 levels and proportion of Y-bearing sperm might be due to diverging exposure profiles among the four populations studied. Furthermore, the consumption of fish (in Sweden) and sea mammals (in Greenland) was the major source of POP exposure. When cohort-to-cohort comparisons were made, the proportion of Y-bearing sperm was higher in these two populations compared with the men from Warsaw and Kharkiv. This discrepancy might be due to differences in PCB-153 levels, but it cannot be excluded that other components in the marine food might affect the Y:X chromosome ratio in sperm.

Another source of difference might be the criteria for selection of the populations included in the study. Whereas the Swedish fishermen are more or less representative for the general population in terms of fertility, the three other cohorts represented proven fertile men. Furthermore, the Greenlandic Inuit are genetically different from the Caucasians. Both factors might, at least to some degree, affect the individual susceptibility to POPs in the deterioration of reproductive function.

Moreover, an increased proportion of Y-bearing sperm in the Swedish cohort compared with a decreased proportion in the Polish cohort might be due to the differing biomarker exposure doses (much higher PCB-153 levels in Sweden compared with those in Warsaw). Indeed, it has been reported that a dose–response relationship of POPs can be biphasic, that is, give a stimulatory response at low doses but an inhibiting response at high doses, which is known as the hormesis phenomenon (Calabrese and Baldwin 1998). Unfortunately, the number of Swedish subjects with levels of PCB-153 exposure corresponding to those in Warsaw was too low to allow an additional subanalysis. Differing results could also be caused by age-dependent differences. Swedish fishermen were on average older compared with the other cohorts (47.1 years in Sweden; 26.1–30.8 years in other populations).

Because of the high correlation between *p*,*p*′-DDE and PCB-153 levels in the Swedish fishermen cohort, it was not possible to disentangle whether the positive associations observed for both compounds with Y-sperm proportion were due to independent effects of these two compounds or the effect of only one of them. When dividing the subjects into four groups according to their PCB-153 and *p*,*p*′-DDE exposure profile, we found slightly higher proportion of Y-bearing sperm in subjects with high PCB-153 and low *p*,*p*′-DDE compared with those with low PCB-153 and high *p*,*p*′-DDE. However, because of strong and probably region-specific associations between the levels of these markers and those of other POPs, we should be cautious in concluding that PCB-153 tended to increase the proportion of Y-bearing sperm but *p*,*p*′-DDE had the opposite effect.

An intriguing question when looking for mechanisms behind the previously reported changes in offspring sex ratio after exposure to POPs is whether there is an association between the sex chromosome distribution in sperm and sex distribution in the offspring. Although the outcome of the pregnancy was known for the three cohorts of fertile men, our study could not give any definite answer to this question because of the insufficient number of subjects and thereby low power of the statistical analysis. This can be illustrated by the number of children fathered by men belonging to the quartiles with the highest and the lowest proportions of Y-bearing sperm; assuming a binomial distribution (boy/girl), the statistical power of an analysis aiming to disclose a change from 53% to 47% of boys fathered was found to be very low (β= 0.07; α= 0.05).

When evaluating the results of the present study, several potential biases need to be considered. The participation rate, in similarity with other semen studies, was low in all cohorts except for the Greenland Inuit. Regarding the Swedish fishermen, the age distributions and the mean number of children were very similar among the participants and the nonparticipants (Rignell-Hydbom et al. 2004). In the three remaining cohorts, time to pregnancy did not differ between those who delivered semen for analysis and those who did not. Therefore, we do not consider selection bias to be of major concern. Moreover, we also believe that residual confounding is probably not an issue of great concern because we considered all potential confounders in the analyses. However, we cannot exclude that imperfect measurements of the confounders caused some residual confounding. Having only a single semen sample could be a limitation. However, there are no data in the literature on possible temporal variation in sex chromosome fractions. Nevertheless, even if such a phenomenon exists, this variability would most likely dilute and not magnify the associations found in the present study.

Another potential bias was the lack of hybridization signal in up to 5% of spermatozoa because of insufficient hybridization. However, a hybridization efficiency of 95% or more is in good accordance with the hybridization efficiency reported by other groups (Johannisson et al. 2002; Martin et al. 1996). We excluded 19% of the cases because of insufficient labeling. However, there was no significant difference considering age, exposure level, and seminal parameters between subjects from whom FISH data were obtained and those excluded from the study. Furthermore, in slightly more than 35% of the slides, < 500 nuclei—the number on which the quality control procedure was based—were scored. Although it might introduce increased imprecision in the methodology, such bias is considered nondifferential and can therefore hardly explain the statistically significant associations found in two of the four cohorts studied.

In conclusion, effects of POP biomarkers on the proportion of Y- and X-bearing sperm found in a cohort of Swedish fishermen were not consistently corroborated in three other study populations. The apparent discrepant findings may be explained by differences in POP exposure profile and dose resulting from variation in lifestyle and diet or they may reflect random variation.

Future epidemiologic studies should include measurements on the bioactivity levels in sera of the exposed individuals as well as a more detailed mapping of the exposure pattern. Although interspecies differences in reproductive response to POPs may exist, experimental studies on laboratory animals might add to our understanding of possible mechanisms of the effect of POP exposure on the sperm sex chromosome status.

## Figures and Tables

**Table 1:** Characteristics of the study populations with respect to exposure and outcome variables, and potential confounders.

	Greenland (*n* = 157)	Sweden (*n* = 149)	Warsaw (*n* = 121)	Kharkiv (*n* = 120)	All (*n* = 547)
Exposure variables					
PCB-153 (ng/g lipid)					
Median	210	200	20	50	90
Mean (95% CI)	350 (270–430)	260 (220–290)	20 (18–24)	55 (43–63)	190 (160–220)
*p*,*p*′-DDE (ng/g lipid)					
Median	590	240	490	1,000	520
Mean (95% CI)	880 (690–1,100)	350 (300–410)	570 (520–640)	1,300 (1,100–1,500)	760 (680–840)
Potential confounders					
Age (years)					
Mean	31	47	30	26	34
Median (min–max)	30 (18.5–51.3)	48 (23.8–67.5)	29 (24.1–46.3)	24 (18.8–40.6)	32 (18.5–67.5)
Current smoking (%)	71.9	23.0	28.3	64.6	57.8
Alcohol, > 21 drinks/week (%)	6.2	—	2.4	—	2.2
Period of abstinence (days)					
Mean	5.7	3.7	6.9	4.1	4.9
Median (min–max)	3.0 (0.5–240)	3.0 (0.5–15)	3.5 (0.5–60)	3.5 (0.5–11)	3.0 (0.5–240)
Outcome variables					
Fraction of Y chromosomes (%)					
Mean	51.2	51.2	50.3	50.7	50.9
Median (min–max)	50.8 (43.2–58.9)	51.1 (47.5–56.7)	50.3 (43.5–53.3)	50.6 (47.1–58.2)	50.7 (43.2–58.9)

Abbreviations: —, not available; max, maximum; min, minimum.

**Table 2:** Crude proportions of Y-bearing spermatozoa according to serum concentrations of PCB-153 and study group.

	Greenland		Sweden		Warsaw		Kharkiv	
PCB-153 range (ng/g lipid)	No.	Mean (min–max)	No.	Mean (min–max)	No.	Mean (min–max)	No.	Mean (min–max)
0–50	9	52.5 (49.2–58.8)	20	50.3 (49.6–51.5)	117	50.3 (48.1–53.2)	68	50.7 (47.2–58.1)
> 50–100	20	51.1 (43.1–58.2)	55	50.8 (47.5–55.3)	3	49.0 (43.5–52.3)	44	50.9 (47.2–55.5)
> 100–200	45	50.9 (48.1–55.2)	52	51.1 (47.5–56.4)	1	50.0 (50.1)	7	50.1 (47.1–52.7)
> 200–400	42	51.5 (47.7–57.8)	19	51.3 (47.6–55.2)	0	—	0	—
> 400	41	51.1 (48.9–56.7)	3	51.6 (48.3–56.7)	0	—	1	49.6 (49.6)

Abbreviations: —, not applicable; max, maximum; min, minimum.

**Table 3:** Crude proportions of Y-bearing spermatozoa according to serum concentrations of *p*,*p*′-DDE and study group.

	Greenland		Sweden		Warsaw		Kharkiv	
*p,p*′-DDE range (ng/g lipid)	No.	Mean (min–max)	No.	Mean (min–max)	No.	Mean (min–max)	No.	Mean (min–max)
0–250	30	51.4 (43.1–58.8)	79	50.7 (47.5–55.3)	9	50.6 (48.9–51.9)	0	—
> 250–500	38	51.1 (48.1–55.2)	40	52.1 (49.3–56.4)	54	50.3 (48.1–52.4)	8	50.2 (49.2–51.0)
> 500–1,000	40	51.4 (48.7–57.8)	21	51.6 (49.2–55.5)	49	50.1 (48.2–52.6)	50	50.9 (47.2–58.1)
> 1,000– 1,500	23	50.7 (47.7–53.5)	7	51.5 (48.3–56.7)	5	51.1 (49.1–53.2)	32	50.7 (48.8–53.6)
> 1,500	26	51.4 (48.9–56.7)	2	50.7 (50.5–51.0)	4	48.9 (43.5–51.4)	30	50.5 (47.1–53.7)

Abbreviations: —, not applicable; max, maximum; min, minimum.

**Table 4:** Effect of PCB-153 (ng/g lipid) and *p*,*p*′-DDE (ng/g lipid) levels in serum (as continuous variable) on proportion of Y-bearing spermatozoa for men from the different populations.

	Percent of Y-sperm			
Variables	No.	β	*p*-Value	95% CI (β)
Ln[PCB-153]				
Greenland	157	0.03^a^^b^^c^^d^	0.83	−0.33 to 0.41
Sweden	149	0.53^a^	0.04	0.001 to 1.05
Warsaw	121	−0.54^a^^a^	0.008	−0.92 to −0.14
Kharkiv	120	0.23^b^	0.27	−0.18 to 0.64
Ln[*p*,*p*′-DDE]				
Greenland	157	0.07^a^^b^^c^	0.65	−0.25 to 0.41
Sweden	149	0.75^a^	< 0.001	0.35 to 1.15
Warsaw	121	−0.35^b^	0.20	−0.89 to 0.19
Kharkiv	120	−0.15	0.54	−0.64 to 0.34

The data were obtained from multiple regression analyses. If the adjusted estimate differed < 10% from the crude estimate, only the crude results are presented. Confounder-adjusted estimated effects (β):

a age;

b abstinence;

c current smoking;

d alcohol consumption.

**Table 5:** Effect of PCB-153 (ng/g lipid) and *p*,*p*′-DDE (ng/g lipid) levels in serum in lowest versus highest category on proportion of Y-bearing spermatozoa for men from the different populations.

	Percent of Y-sperm			
Variables	No.	β	*p*-Value	95% CI (β)
Ln[PCB-153]				
Greenland	157	−0.95^a^	0.227	−2.51 to 0.60
Sweden	149	1.56^a^^b^^c^	0.192	−0.68 to 3.82
Warsaw	121	NA	—	—
Kharkiv	120	NA	—	—
Ln[*p*,*p*′-DDE]				
Greenland	157	0.42^a^^b^	0.477	−0.75 to 1.61
Sweden	149	0.03^a^^b^^c^	0.980	−2.31 to 2.36
Warsaw	121	−1.47^a^^b^	0.064	−3.04 to 0.08
Kharkiv	120	NA	—	—

—, not available; NA, not analyzed because data were not available. The data were obtained from multiple regression analyses. If the adjusted estimate differed < 10% from the crude estimate, only the crude results are presented. Confounder-adjusted estimated effects (β):

a age;

b abstinence;

c current smoking;

d alcohol consumption.
